# Supplementary material for: The Effects of Smoking on Telomere Length, Induction of Oncogenic Stress, and Chronic Inflammatory Responses Leading to Aging
Source: Cells. 2024 May 21;13(11):884. doi: 10.3390/cells13110884 (PMC11172003; doi:10.3390/cells13110884)
Supplement: Supplementary file 1 [file cells-13-00884-s001.zip › cells-2917109-supplementary.pdf]

**Supplementary Materials**

**TABLE S1. DEMOGRAPHICS FOR SERUM AND BUCCAL SAMPLES OF 20 NON-SMOKERS AND 11 SMOKERS**

| | Sex | | Race | Age | | |
|---|---|---|---|---|---|---|
| | Male | Female | Caucasian | 50-59 | 60-69 | ≥70 |
| Non-Smokers | 11 | 9 | 20 | 1 | 11 | 8 |
| Smokers | 7 | 4 | 11 | 6 | 3 | 2 |

**TABLE S2. DEMOGRAPHICS FOR LUNG TISSUE SECTION OF 22 NON-SMOKERS AND 24 SMOKERS**

| | Sex | | Race | | Age | | |
|---|---|---|---|---|---|---|---|
| | Male | Female | Caucasian | African American | 40-59 | 60-79 | ≥80 |
| Non-Smokers | 8 | 14 | 18 | 4 | 7 | 13 | 2 |
| Smokers | 16 | 8 | 20 | 4 | 11 | 13 | 0 |

**TABLE S3. DEMOGRAPHICS FOR LUNG TISSUE SECTIONS OF ISG15 WITH 20 NON-SMOKERS AND 22 SMOKERS**

| | Sex | | Race | | Age | | | |
|---|---|---|---|---|---|---|---|---|
| | Male | Female | Caucasian | African American | 40-59 | 60-79 | ≥80 | Unknown |
| Non-Smokers | 6 | 14 | 17 | 3 | 6 | 11 | 2 | 1 |
| Smokers | 15 | 7 | 22 | 0 | 10 | 12 | - | - |

**FIGURE S1. GENE EXPRESSION LEVELS IN 11 SMOKERS AND 11 NON-SMOKERS**

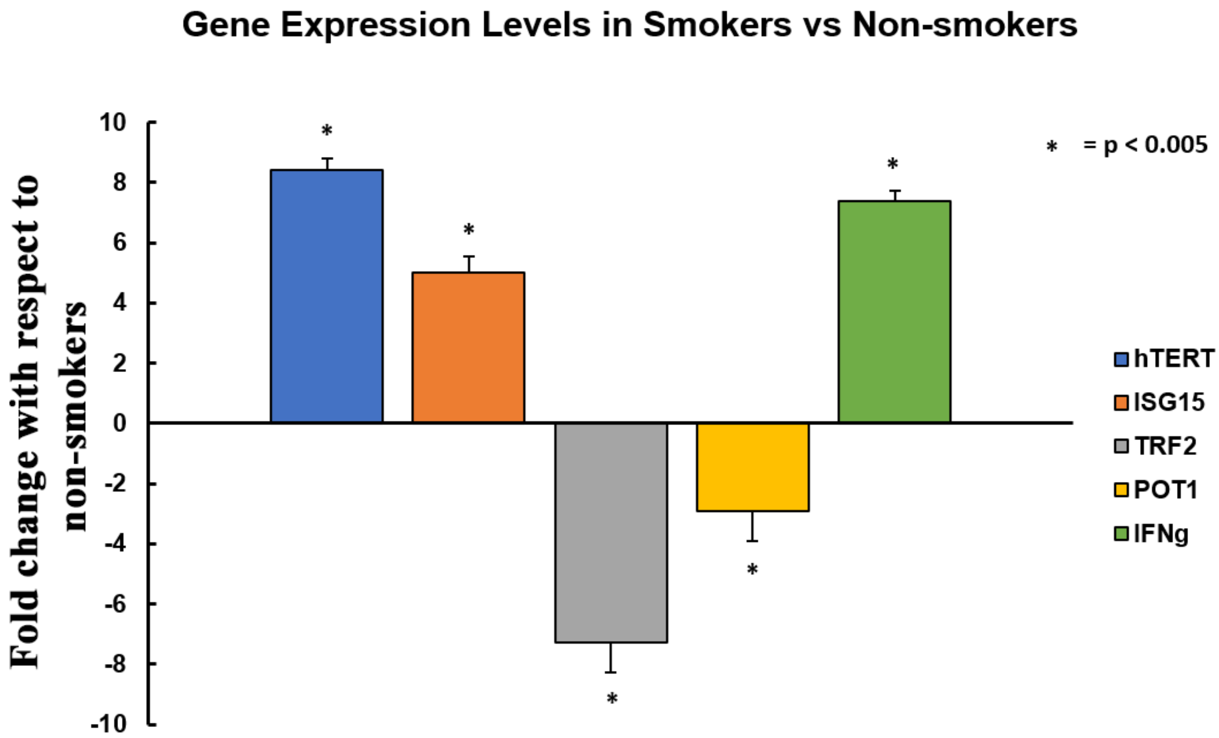


**FIGURE S1. Comparison of gene expression levels of hTERT, ISG15, TRF2, and POT1 in 11 smokers vs. 11 non-smokers using qPCR.** Whole blood of 11 smokers and 11 non-smokers was processed to isolate leukocytes. Total RNA was extracted from blood leukocytes and was quantified and analyzed using qPCR for mRNA levels of genes of interest. The data was normalized with GAPDH, and graphical representation is relative to expression of respective genes in smokers with respect to non-smokers. The data was collected from triplicates (n=3), and the result was statistically significant by two tailed t-test analysis.

**FIGURE S2. PERCENT METHYLATION OF hTERT IN 11 SMOKERS AND 11 NON-SMOKERS**

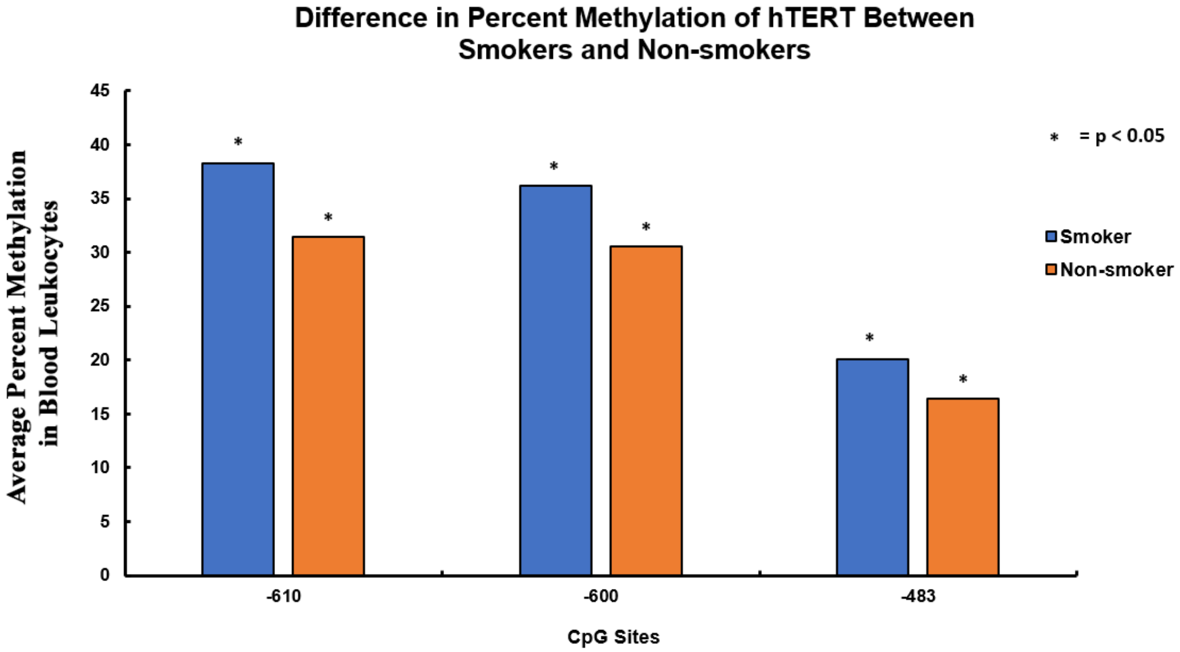


**Figure S2. Percent methylation analysis of CpG sites in the promoter region of hTERT between smokers and non-smokers.** Genomic DNA was isolated from the blood leukocytes and buccal epithelial cells of smokers and non-smokers. This DNA was then bisulfite converted and methylation specific PCR was conducted. The PCR product was run on an agarose gel and validated after which the PCR product was sent for Next Generation Sequencing. Statistical analysis was performed by way of a two-tailed independent t-tests.

**FIGURE S3. TELOMERE LENGTH OF BLOOD LEUKOCYTES IN 11 SMOKERS AND 11 NON-SMOKERS**

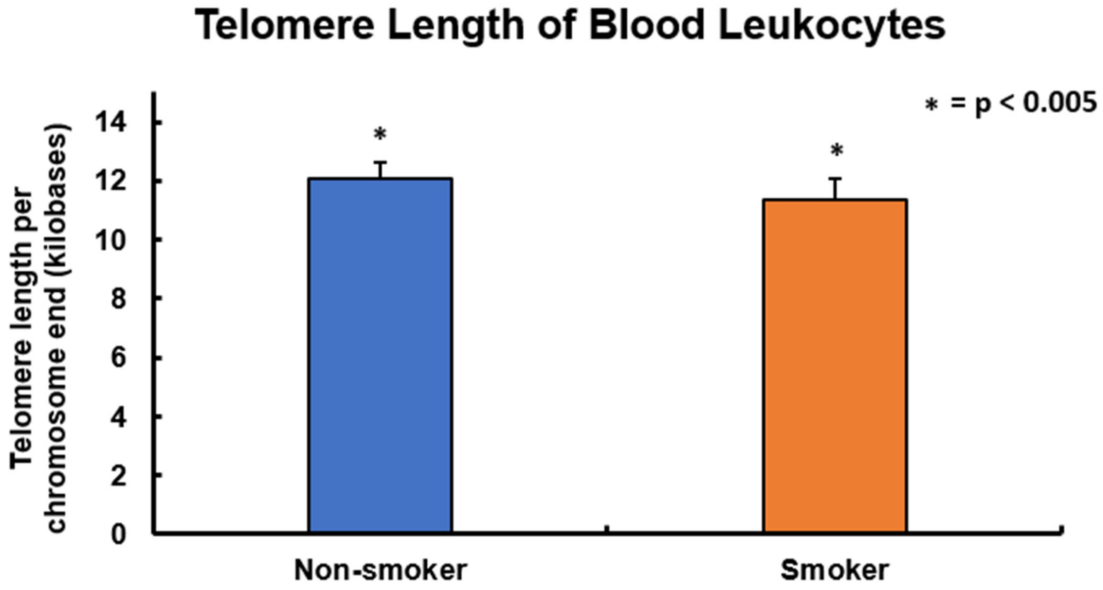


**Figure S3. Comparing telomere length of blood leukocytes in 11 smokers and 11 non-smokers.** Whole blood of 11 smokers and 11 non-smokers was processed to isolate leukocytes. Isolated genomic DNA was quantified and analyzed in a telomere length assay to determine the relative telomere length per chromosome end. The data was normalized using assay reference primer set and was found to be statistically significant by two tailed t-test analysis.

**FIGURE S4. TELOMERE LENGTH OF BUCCAL EPITHELIAL CELLS IN 11 SMOKERS AND 11 NON-SMOKERS**

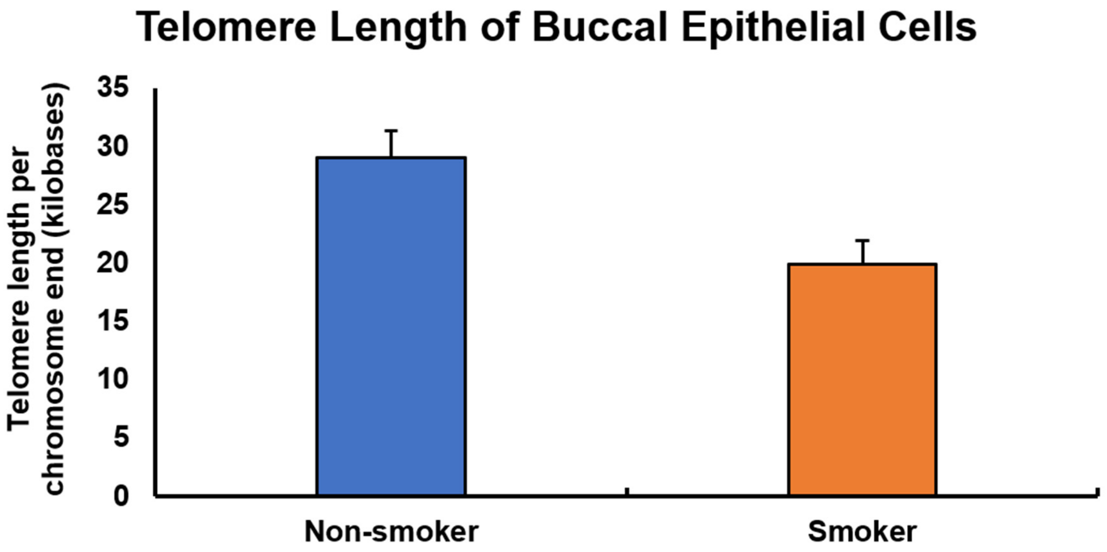


**Figure S4. Comparing telomere length of buccal epithelial cells in 11 smokers and 11 non-smokers.** Buccal swabs of 11 smokers and 11 non-smokers was processed to isolate buccal epithelial cells. Isolated genomic DNA was quantified and analyzed in a telomere length assay to determine the relative telomere length per chromosome end. The data was normalized using assay reference primer set and was found to be statistically significant by two tailed t-test analysis.
